# Drivers determining tuberculosis disease screening yield in four European screening programmes: a comparative analysis

**DOI:** 10.1183/13993003.02396-2022

**Published:** 2023-10-12

**Authors:** Dominik Zenner, Daniella Brals, Joanna Nederby-Öhd, Dee Menezes, Robert Aldridge, Sarah R. Anderson, Gerard de Vries, Connie Erkens, Valentina Marchese, Alberto Matteelli, Morris Muzyamba, Job van Rest, Ineke Spruijt, John Were, Giovanni Battista Migliori, Knut Lönnroth, Frank Cobelens, Ibrahim Abubakar

**Affiliations:** 1 Faculty of Population Health Sciences, University College London, London, UK; 2 Wolfson Institute of Population Health, Queen Mary University of London, London, UK; 3 Amsterdam University Medical Centers, location University of Amsterdam, Department of Global Health, Amsterdam, The Netherlands; 4 Amsterdam Public Health, Global Health, Amsterdam, The Netherlands; 5 Department of Global Public Health, Karolinska Institutet, Stockholm, Sweden; 6 Institute of Health Informatics Research, University College London, London, UK; 7 UK Health Security Agency, London, UK; 8 National Institute for Public Health and the Environment, Bilthoven, The Netherlands; 9 KNCV Tuberculosis Foundation, The Hague, The Netherlands; 10 WHO Collaborating Center for TB/HIV and the TB Elimination Strategy, University of Brescia, Brescia, Italy; 11 Servizio di Epidemiologia Clinica delle Malattie Respiratorie, Istituti Clinici Scientifici Maugeri IRCCS, Tradate, Italy

## Abstract

**Background:**

The World Health Organization End TB Strategy emphasises screening for early diagnosis of tuberculosis (TB) in high-risk groups, including migrants. We analysed key drivers of TB yield differences in four large migrant TB screening programmes to inform TB control planning and feasibility of a European approach.

**Methods:**

We pooled individual TB screening episode data from Italy, the Netherlands, Sweden and the UK, and analysed predictors and interactions for TB case yield using multivariable logistic regression models.

**Results:**

Between 2005 and 2018 in 2 302 260 screening episodes among 2 107 016 migrants to four countries, the programmes identified 1658 TB cases (yield 72.0 (95% CI 68.6–75.6) per 100 000). In logistic regression analysis, we found associations between TB screening yield and age (≥55 years: OR 2.91 (95% CI 2.24–3.78)), being an asylum seeker (OR 3.19 (95% CI 1.03–9.83)) or on a settlement visa (OR 1.78 (95% CI 1.57–2.01)), close TB contact (OR 12.25 (95% CI 11.73–12.79)) and higher TB incidence in the country of origin. We demonstrated interactions between migrant typology and age, as well as country of origin. For asylum seekers, the elevated TB risk remained similar above country of origin incidence thresholds of 100 per 100 000.

**Conclusions:**

Key determinants of TB yield included close contact, increasing age, incidence in country of origin and specific migrant groups, including asylum seekers and refugees. For most migrants such as UK students and workers, TB yield significantly increased with levels of incidence in the country of origin. The high, country of origin-independent TB risk in asylum seekers above a 100 per 100 000 threshold could reflect higher transmission and re-activation risk of migration routes, with implications for selecting populations for TB screening.

## Tweetable abstract

**Factors** **associated with TB screening yield included increasing age, migrant typology, TB incidence in country of origin, TB case contact and period of screening. The TB yield among asylum seekers was higher than for other migrant categories.** https://bit.ly/3VMLwFp

## Introduction

With 1.6 million annual fatalities, tuberculosis (TB) is a leading cause of death from any infectious agent globally [1]. A combination of biological and well-recognised socioeconomic risk factors makes TB a complex disease to control, necessitating the use of a wide range of TB control mechanisms. The multipronged approach is reflected in the World Health Organization End TB Strategy (2016–2035) [2] and the Sustainable Development Goals, aiming to decrease incidence, deaths and catastrophic costs through adoption of wide-ranging measures [3]. This includes screening of migrants from high-incidence countries among other groups, which is considered key to achieving TB elimination in low-incidence countries [4–6]. In 2020, progress to reach the End TB Strategy targets was disrupted by the coronavirus disease 2019 (COVID-19) pandemic. Disrupted TB services [7, 8], affecting all parts of national TB control programmes, led to decreases in TB notifications, with an expected significant and observed increase of TB mortality over the next few years [1, 9]. The “path to recovery” will require emphasis on early diagnosis [10] and thereby increase the relevance of screening.

Screening programmes for TB disease have a long history, including the radiographic screening in the early 20th century, mostly stopped as a population-wide approach with decreasing incidence and (cost-)effectiveness [11]. Nonetheless, this approach continues to be used for specific risk groups, including people from high TB risk countries migrating to low TB incidence countries [12, 13].

Our recent study, which described four migrant screening programmes in Europe using the same database, showed that in addition to considerable programme-level variation there was individual-level variation in TB screening yield, driven by age, TB incidence in the country of birth and migrant group [14]. While individual risk factors have been previously described, more granular analysis is needed to understand the relative importance of these variables and how they interact to determine TB yield. Risk variations might apply to all in a similar fashion or differently to different population groups. In addition, the interplay between programmatic and individual variables is poorly understood. Such knowledge will enable better understanding and decision making about whether and how much programmes can or should be harmonised or not, considering country differences in their populations or policy preferences. In addition, no previous studies are available reporting on merged large individual datasets from different European countries and this therefore potentially represents a pilot study to improve European TB screening surveillance.

Therefore, in our European Commission-funded study, coordinated with the European Centre of Disease Prevention and Control (ECDC), we analysed key drivers behind similarities and differences in TB yield between different migrant screening programmes and determined the relative importance of these factors, including demographic characteristics, specific programme features and year of screening, as well as the role of interaction between these characteristics.

## Methods

We compared individual-level data from four European TB screening programmes pooled in a multicountry database. The often chest radiography-based screening programmes have been previously described [15, 16]. The database contains data from the national data registry on new immigrants in the Netherlands [17], the Swedish Migration Authority and electronic medical records in the Stockholm Region health services in Sweden [18], specific district screening projects in Italy, and the UK new entrant screening programme [19], with screening records from 2005 to 2018 [15]. We also conducted surveys and semistructured interviews to obtain contextual and programme-level information to assist in data interpretation [14].

Following explorative analysis with simple cross-tabulations and graphics (data not shown), we performed univariable and multivariable logistic regression analyses to determine the effect of individual-level (*e.g.* demographic) and programme-level exposure variables on TB yield. We defined yield (primary outcome) as prevalent cases, notified within 151 days of screening over the total screened population using a modified version of the European Union TB case definition and consistent with the Dutch definition of prevalent cases, and included stratification into possible, probable and confirmed cases (supplementary material) [16]. Recent self-reported (*via* screening questionnaire) household-type TB contact was included in the analysis, where available. We also analysed the effect of exposure variables on a range of secondary outcomes, including patients with any lesions compatible with TB on chest radiography (chest radiography positivity) and patients with a positive TB culture result.

There are different categories and levels for potential predictors of TB yield, including individual risk (*e.g.* patient demography), higher-level programmatic or country interventions, time-bound factors and predictors on the screening pathway.

Variables were deemed fit for inclusion in the multivariable model if they had a p-value <0.2 in univariate analysis and/or had *a priori* biological plausibility for outcome association. The logistic regression model was built manually by including new variables in a stepwise fashion, assessing the explanatory power of each new variable for TB yield through a likelihood ratio test (LRT) and the change of effect estimates in the model. The relationship between these factors was explored first by mapping (*e.g.* through direct acyclic graphs) and cross-tabulation. We tested violations of assumptions of independence of variables formally through correlation coefficients and the variance inflation factor. If collinearity was detected, only the variable considered most informative and with higher biological plausibility was retained for further analysis.

New variables were kept in the model if they significantly added to the explanatory power of the model (LRT p<0.05), changed effect estimates and/or there was *a priori* biological plausibility, such as age and sex. We tested continuous variables for linear association (Chi-squared test for trend) and treated them as categorical if they were not linear.

We have previously demonstrated significant variation at the individual (largely demographic, risk factor dependent) and screening programme level [14], and we sought to fit a hierarchical multilevel regression model to explore the effect of individual predictors as well as the effect of programme-level predictors on TB yield. The collinearity of key programme-level indicators, such as the partial collinearity between migrant typology with screening programmes, precluded hierarchical analysis of the full dataset. We therefore performed logistic regression analysis instead, using robust standard error estimation, adjusted for clustering at the TB screening programme.

Through restriction of records to asylum seekers, we performed a subgroup analysis with screening records in three of the four programmes in a hierarchical multilevel model, adjusting for the higher level of the programme variable with individuals as the unit of analysis, nested in the higher level of the screening programme.

Variables with a biological plausibility of effect modification were tested by assessing interaction terms using an LRT. We used the Stata lincom command for post-estimation of effect differences in the models. MS Excel for Mac version 16.46 (Microsoft, Redmond, WA, USA) was used for figures and tables. All statistical analysis was carried out with Stata version 16.1 (StataCorp, College Station, TX, USA).

## Results

**FIGURE 1:**
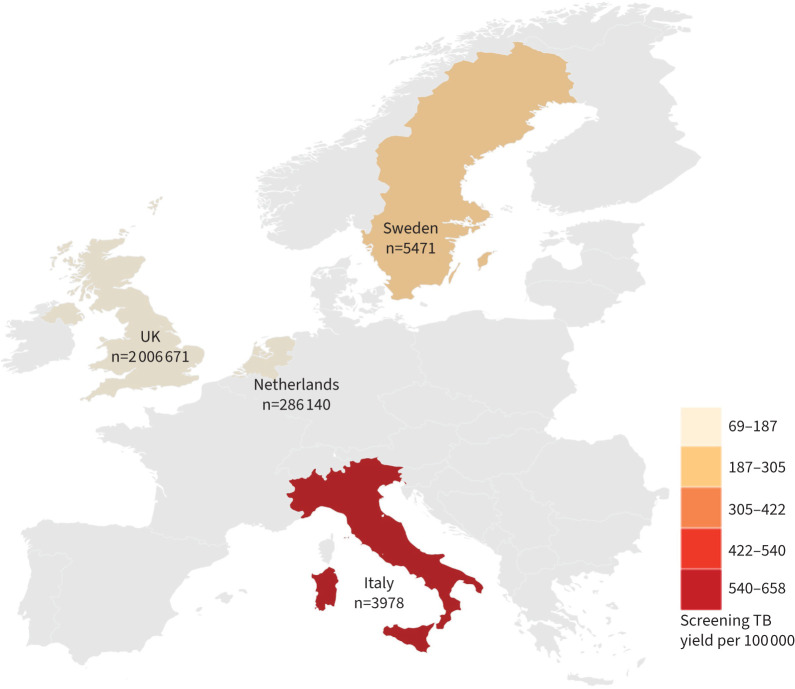
Yield from tuberculosis (TB) screening (colours) and total number of screens (numbers) performed by screening programmes.

We included 2 302 260 screening episode records between 2005 and 2018 for 2 107 016 migrants to four European countries; of these, 3978 episodes were reported from Italy, 286 140 from the Netherlands, 5471 from Sweden and the remaining 2 006 671 from the UK (figure 1). In total, the programmes detected 1658 persons with TB (26 from Italy, 238 from the Netherlands, 11 from Sweden and 1383 from the UK) [14].

In our logistic regression model, older age in adults, migrant typology, greater TB incidence in the country of origin, being a contact of a TB case, and the screening years 2010–2012 and 2013–2015 were significantly associated with a higher TB yield in both univariate and multivariate regression analysis, adjusted for clustering at the programme level (main model) (table 1). Asylum seekers and individuals who came on settlement and family visas, as well as immigrants to the Netherlands, had a significantly higher TB yield compared with UK-bound migrants on student or worker visas. TB yield also varied significantly over time, with lower yields in early and more recent years.

**TABLE 1:** Logistic regression model assessing predictors for tuberculosis (TB) at the time of screening (prevalent TB or yield)

	**Population (n** **)**	**TB cases (n)**	**Univariable analysis** **OR (95% CI)**	**Multivariable analysis** **adjusted OR (95% CI)**	**p-value**
**Sex**					0.30
Female	955 531	751	Reference		
Male	1 058 396	790	0.95 (0.86–1.05)	1.08 (0.96–1.23)	
**Age group**					<0.01
<18 years	248 422	100	0.52 (0.42–0.63)	0.36 (0.22–0.57)	
18–34 years	1 485 035	1157	Reference		
35–54 years	251 796	197	1.00 (0.86–1.17)	0.89 (0.81–0.98)	
≥55 years	140 810	125	1.14 (0.95–1.37)	2.91 (2.24–3.78)	
**Migrant typology**					<0.01
UK students and workers	1 056 195	572	Reference		
Netherlands immigrants	163 116	101	1.14 (0.93–1.41)	0.79 (0.30–2.07)	
Asylum seekers (Italy, Netherlands, Sweden)	132 198	174	2.43 (2.05–2.88)	3.19 (1.03–9.83)	
UK settlements and family	576 485	624	2.00 (1.78–2.24)	1.78 (1.57–2.01)	
UK working holiday and others	112 558	81	1.33 (1.05–1.68)	1.06 (0.88–1.27)	
**Incidence in country of origin, per 100 000^#^**					<0.01
<50	146 946	39	Reference		
50–100	504 131	122	0.91 (0.64–1.31)	2.12 (0.88–5.06)	
100–200	460 651	397	3.25 (2.34–4.51)	7.52 (5.82–9.71)	
200–300	808 274	692	3.23 (2.34–4.45)	6.68 (5.56–8.02)	
<300	251 040	348	5.22 (3.75–7.27)	19.78 (15.86–24.67)	
**Contact of TB case^¶^**					<0.01
No	1 917 818	1287	Reference		
Yes	2986	43	21.46 (15.80–29.14)	12.25 (11.73–12.79)	
Unknown	379 798	328	1.29 (1.14–1.45)	1.49 (0.58–3.86)	
**Time period of screening**					<0.01
Before 2010	370 604	262	Reference		
2010–2012	336 007	309	1.30 (1.10–1.53)	1.27 (0.97–1.67)	
2013–2015	717 099	669	1.32 (1.14–1.52)	1.55 (1.49–1.60)	
2016–2018	876 892	418	0.67 (0.58–0.79)	0.77 (0.54–1.11)	

Standard errors were adjusted for clustering at the programme level. ^#^: incidence in the country of origin refers to 2019 World Health Organization estimates [20]; ^¶^: TB contacts are either family or other close (household-type) contacts. p-values calculated using the likelihood ratio test comparing to restricted models with less predictors.

We found significant interaction between TB incidence in the country of origin and migrant typology (p<0.001) (supplementary table S5). Within most migrant categories such as UK students and workers, TB yield significantly increased by incidence category, albeit at different levels. TB yield among asylum seekers was higher than for any other migrant category, but where their country of origin had an incidence of above 100 per 100 000, TB yield did not increase further. We also found significant interactions between TB yield determined by being a TB contact and country of origin (p<0.003) and age and migrant typology (supplementary tables S1 and S2).

We replicated the model with two secondary outcomes. Restricting the analysis to culture-confirmed TB cases (n=1278 (77.1% of all cases)) (supplementary table S3) leads to different effect estimates, most notably for migrant typology, but with an overall direction of effect similar compared with the main model.

We also replicated the model analysing TB-related abnormalities on chest radiography. This is used as a first screening step in many of the programmes leading to a selection of individuals for further tests with higher specificity (*e.g.* sputum culture). The multivariable analysis showed a similar effect for most variables, but a smaller effect of TB incidence in the country of origin and migrant typology, and a larger effect of age on chest radiography abnormality, compared with the main model (supplementary table S4).

Finally, we performed an analysis on asylum seekers only among the three programmes where data was available, to allow adjustment for programme level and in-depth analysis of asylum seeker characteristics associated with TB yield. There were 132 372 screened asylum seekers recorded in total (3978 from Italy, 122 923 from the Netherlands and 5471 from Sweden). Within this group, there were 174 TB cases (26 from Italy, 137 from the Netherlands and 11 from Sweden), giving yields of 131.1 (95% CI 113.3–152.5) per 100 000 overall, and 653.6 (95% CI 445.4–958.2), 111.5 (95% CI 94.3–131.8) and 201.1 (95% CI 111.4–362.7) per 100 000 for Italy, the Netherlands and Sweden, respectively. In total, 123 (70.7%) of the 174 TB cases were culture confirmed: 18 (69.2%) of those detected in Italy, 98 (71.5%) in the Netherlands and seven (63.6%) in Sweden.

In the simple multivariable logistic regression model restricted to asylum seekers (table 2), the differences between the screening programmes became more apparent. Asylum seekers were almost three and more than five times as likely to be diagnosed with TB in Sweden and Italy, respectively, compared with the Netherlands after adjustment for other factors. Notably, the difference in TB yields by incidence in the country of origin among asylum seekers was only significant in lower incidence categories (OR 5.07 (95% CI 3.12−8.2); p<0.001, comparing <50 and 50–100 per 100 000) but not in higher categories (OR 0.97 (95% CI 0.53–1.78); p=0.91, comparing 200–300 and >300 per 100 000). A similar observation can be made for age groups, with significant differences in categories between the youngest but not older age groups. We tested but did not detect significant interactions in this restricted model.

**TABLE 2:** Logistic regression model restricted to asylum seekers^#^ assessing determining factors for prevalent tuberculosis (TB) at the time of screening (yield)

	**Population (n)**	**TB cases (n)**	**Univariable analysis** **OR (95% CI)**	**Multivariable analysis** **adjusted OR (95% CI)**	**p-value**
**Programme**					<0.001
Netherlands	122 786	137	Reference		
Sweden	5460	11	1.81 (0.98–3.34)	2.86 (1.38–5.91)	
Italy	3952	26	5.90 (3.87–8.98)	5.25 (3.05–9.05)	
**Sex**					0.007
Female	47 409	40	Reference		
Male	84 677	134	1.88 (1.32–2.67)	1.70 (1.18–2.44)	
**Age group**					0.149
<18 years	43 843	39	0.52 (0.36–0.75)	0.65 (0.44–0.96)	
18–34 years	60 955	104	Reference		
35–54 years	22 985	26	0.66 (0.43–1.02)	0.99 (0.63–1.53)	
≥55 years	3815	4	0.61 (0.23–1.67)	0.95 (0.35–2.61)	
**Incidence in country of origin, per 100 000^¶^**					<0.001
<50	58 810	25	Reference		
50–100	27 859	48	4.05 (2.50–6.57)	5.12 (3.13–8.36)	
100–200	17 440	52	7.01 (4.35–11.30)	6.12 (3.71–10.10)	
200–300	11 872	34	6.74 (4.02–11.30)	5.54 (3.20–9.59)	
<300	5077	15	6.95 (3.66–13.19)	7.52 (3.91–14.47)	
**Time period of screening**					<0.001
2010–2012	19 392	41	Reference		
2013–2015	81 138	99	0.58 (0.40–0.83)	0.55 (0.38–0.81)	
2016 and after	31 668	34	0.51 (0.32–0.80)	0.16 (0.09–0.29)	

Standard errors were adjusted for clustering at the programme level. ^#^: analysable records only (some records were excluded due to missing data); ^¶^: incidence in country of origin refers to 2019 World Health Organization estimates [20]. p-values calculated using the likelihood ratio test comparing to restricted models with less predictors.

A hierarchical model with two levels (random intercept/fixed slope), adjusting for the screening programme as the higher level (table 3), showed a better fit of the data than the simple logistic regression model (LRT p<0.001), and effects were different for sex (males now have significantly larger risk) with similar effects for age, incidence in the country of origin and time period of screening.

**TABLE 3:** Restricted (to asylum seekers^#^) multilevel (random slope, random intercept) regression model assessing determining factors for prevalent tuberculosis at the time of screening (yield)

	**Adjusted OR (95% CI)**	**p-value (Wald)**	**p-value (LRT)**
**Sex**			<0.01
Female	Reference		
Male	1.72 (1.20–2.47)	<0.01	
**Age group**			<0.01
<18 years	0.66 (0.45–0.98)	0.04	
18–34 years	Reference		
35–54 years	1.00 (0.64–1.56)	0.99	
≥55 years	1.02 (0.37–2.78)	0.98	
**Incidence in country of origin, per 100 000^¶^**			<0.01
<50	Reference		
50–100	5.76 (3.53–9.40)	<0.01	
100–200	6.71 (4.08–11.04)	<0.01	
200–300	6.58 (3.83–11.30)	<0.01	
<300	9.14 (4.78–17.48)	<0.01	
**Time period of screening**			<0.01
2016	Reference		
2015 and before	2.14 (0.32–14.47)	0.44	
2017 and after	0.41 (0.07–2.46)	0.33	
**Programme (cluster effect)**	3.04 (0.43–21.63)		<0.01
**Time period (random slope)**	3.10 (0.44–21.58)		

LRT: likelihood ratio test. ^#^: analysable records only (some records were excluded due to missing data); ^¶^: incidence in the country of origin refers to 2019 World Health Organization estimates [20].

## Discussion

We analysed relevant exposure factors for TB in a pooled database of four large TB disease screening programmes in Europe, and found that age, male sex, screening period and having been a contact to a TB case are important risk factors for TB, and demonstrated increasing TB risk with increasing incidence in the country of origin. Our analyses show that immigrants to the Netherlands and those on UK settlement and dependant visas, and particularly asylum seekers, are significantly more likely to be detected with TB compared with UK students and workers.

Our findings compare well to the literature, including the association of TB yield with age, male sex, having had TB contact, increasing TB risk with higher incidence in the country of origin [17, 21–23] and migrant typology [24] as previously described, mostly in country-specific studies [17, 25, 26]. In addition, our large sample allowed us to show significant interactions between different key factors often used to determine eligibility for TB screening programmes.

In our cross-country comparison, we demonstrate that migrant typology is a significant risk factor, particularly being an asylum seeker. However, from a threshold of 100 per 100 000 further country of origin incidence increases do not significantly change TB risk in this group. This was confirmed by subanalysis restricted for incidence in the country of origin. Although the higher TB risk among asylum seekers has been described in country-specific studies, there TB risk often increases alongside country of origin incidence [25].

Country of origin-independent risk among asylum seekers is likely related to unknown or unmeasured cofactors and could relate to specific circumstances asylum seekers face during their journeys or on arrival, rather than a reflection of background TB incidence in their country of origin. While the migration experience in the movement phase varies by route, length of time and socioeconomic circumstances, health hazards are well documented, particularly along the Mediterranean route [27–29]. Routes of migration may include long stays in third countries, alongside economic hardship or specific hazards such as imprisonment [28], in turn increasing TB risk through re-activation or exposure to high-transmission environments, such as overcrowded accommodations or prisons [30]. Lack of access to healthcare during the journey may decrease detection and worsen TB outcomes. Note that lack of healthcare access has likely worsened for migrants during the recent COVID-19 pandemic [31].

Genomic evidence has previously helped to document increased TB risk along migration routes and the migration route may in part explain the high TB detection rate in Italy [32]. The TB risk en route raises important considerations well beyond the central Mediterranean route [33]. More in-depth studies are required to describe the TB risk along the route.

The finding that, compared with other migrant typologies, TB risk among asylum seekers from high-incidence countries of origin was less dependent on the country of origin is important for determining eligible populations for TB screening programmes.

**FIGURE 2:**
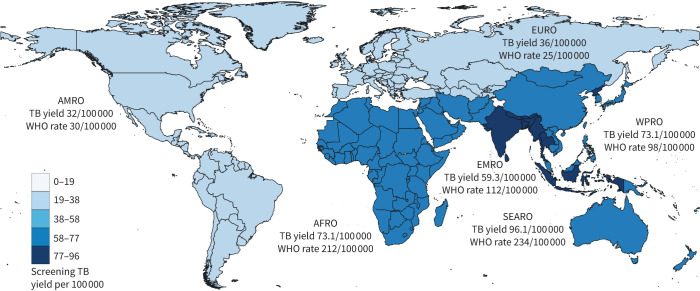
Yield from tuberculosis (TB) screening and World Health Organization (WHO) estimated TB incidence rate (2021) in WHO world regions [1]: African Region (AFRO), Region of the Americas (AMRO), Eastern Mediterranean Region (EMRO), European Region (EURO), South-East Asia Region (SEARO) and Western Pacific Region (WPRO). Colours denote TB yield.

Some programmes have started to apply differential screening criteria to different migrant groups [34]. In the Netherlands, evaluation of national screening data led to adjustments [17], including stopping the screening of immigrants from countries of origin with a TB incidence below 100 per 100 000 and of asylum seekers from countries of origin with a TB incidence below 50 per 100 000 [35]. Practice remains highly variable in Europe due to epidemiological and policy considerations, but possibly also linked to a scarcity of evidence on how TB risk varies by population and the migration journey [34]. The increased risks during migration journeys may warrant additional healthcare provisions for asylum seekers on arrival and screening programmes may benefit from including all asylum seekers from countries with a TB incidence above 100 per 100 000 (figure 2).

TB risk among asylum seekers was less dependent on demographic factors such as age, although it was associated with male sex, which could reflect poorly explained, but well-established global prevalence patterns or slightly different migration patterns, with single male migration frequently predating family reunions, and merits further research [36].

We also demonstrated significant differences in yield between different screening programmes, which might be explained by different compositions of the migrant population or by different algorithms and settings. Since the former was at least partly adjusted for in the model, the latter is likely more relevant. Evidence from stakeholder interviews as well as the country-specific studies [17, 21, 25, 34] from screening programmes demonstrate considerable differences in their scope, timing, target population, algorithm and setting, which taken together can explain some of the observed variation in screening yield. For example, in the Netherlands’ programme, asylum seekers are screened within 24 h of entry, while immigrants are screened up to 3 months later.

The impact of the screening test [37], setting and algorithm [38] have been previously discussed, and these affect TB yield independent of demographic risk factors of the screening cohort. The effects of increasing age and close TB contact on TB risk have previously been documented [39]. We confirmed this in our overall screening cohort.

There are strengths and limitations in our observational study which utilises data collected for programmatic reasons and subject to variable recording quality. Our analysis benefits from the ability to directly compare individual records in different European countries. Harmonisation of variables across programmes may have led to loss of granularity for some variables, such as age, which had to be reclassified as categorical. Migrant programmes are often based on legal frameworks and in the case of the UK are linked to border management, therefore overall data quality was relatively high and misclassification for exposures and outcomes rare. There was some missing data [14] on a few exposure variables, likely missing at random, which in complete case analysis slightly decreased the sample size but in our large dataset unlikely affected power and conclusions. The fact that the analysis of probable and confirmed cases is highly compatible with the analysis of microbiologically confirmed cases further minimises the likelihood of outcome misclassification. Analysis of chest radiography abnormalities was also compatible with the main results and showed expected differences to the main model, *e.g.* an accentuated yield increase with increasing age, likely due to the decreased specificity of the screening tool since age may increase the likelihood of chest radiography abnormalities, which on further investigation turn out not to be TB related.

This first attempt to merge individual data from screening monitoring systems of four different European countries also represents a feasibility study for a future evolution of the ECDC surveillance systems.

In conclusion, our comparative analysis of four large migrant TB screening programmes confirmed the applicability of many previously known TB risk factors and provided more evidence about their effect size and the interactions between them, particularly for migrant typology and incidence in the country of origin. Traditionally, programmes have used relatively simple, often unidimensional eligibility criteria for screening, but some have argued for more complex or even bespoke risk algorithms, particularly in respect of screening for TB infection [40]. A similar argument could be built for TB disease and our research provided more detail on risk interactions which could guide this process. On the other hand, the finding that asylum seekers have an increased TB risk, which was less affected by country of origin and age, raises the importance of TB risk during the migration journey. This elevated risk in asylum seekers requires urgent research and is an important point of enquiry for practitioners in first reception centres. Widening programme eligibility criteria may make sense for asylum seekers.

Our findings could be used to refine screening policy recommendations, which may take the differential effects of risk factors into consideration to optimise programmes, including their effectiveness and cost-effectiveness. Going forward, it will be good to make progress harmonising screening criteria and programmes across Europe through regional TB control recommendations and, eventually, a future upgrade of European TB surveillance systems, to allow close monitoring of screening outcomes at the country and regional level.

## Supplementary material

10.1183/13993003.02396-2022.Supp1 **Please note:** supplementary material is not edited by the Editorial Office, and is uploaded as it has been supplied by the author.
Supplementary material ERJ-02396-2022.Supplement

## Shareable PDF

10.1183/13993003.02396-2022.Shareable1 This one-page PDF can be shared freely online.
Shareable PDF ERJ-02396-2022.Shareable
