# Supplementary material for: Drivers determining tuberculosis disease screening yield in four European screening programmes: a comparative analysis
Source: Eur Respir J. 2023 Oct 12;62(4):2202396. doi: 10.1183/13993003.02396-2022 (PMC10568038; doi:10.1183/13993003.02396-2022)
Supplement: Supplementary file 1 [file ERJ-02396-2022.Supplement.pdf]

Drivers determining TB disease screening yield in four European screening programmes: a comparative analysis - Annex

EU Tuberculosis case definition(1)

Clinical Criteria

Any person with the following two:
— Signs, symptoms and/or radiological findings consistent with active tuberculosis in any site

AND
— A clinician's decision to treat the person with a full course of anti-tuberculosis therapy OR

A case discovered post-mortem with pathological findings consistent with active tuberculosis that would have indicated anti-tuberculosis antibiotic treatment had the patient been diagnosed before dying

Laboratory Criteria

Laboratory criteria for case confirmation

At least one of the following two:

— Isolation of Mycobacterium tuberculosis complex (excluding Mycobacterium bovis-BCG) from a clinical specimen

— Detection of Mycobacterium tuberculosis complex nucleic acid in a clinical specimen AND positive microscopy for acid-fast bacilli or equivalent fluorescent staining bacilli on light microscopy

Laboratory criteria for a probable case

At least one of the following three:
— Microscopy for acid-fast bacilli or equivalent fluorescent staining bacilli on light microscopy — Detection of Mycobacterium tuberculosis complex nucleic acid in a clinical specimen
— Histological appearance of granulomata
Epidemiological Criteria NA

Case Classification

1. Possible case
   Any person meeting the clinical criteria
2. Probable case
   Any person meeting the clinical criteria and the laboratory criteria for a probable case
3. Confirmed case
   Any person meeting the clinical and the laboratory criteria for case confirmation

Antimicrobial resistance

The results of antimicrobial susceptibility tests must be reported according to the methods and criteria agreed between ECDC and Member States as specified by the European Reference Laboratory Network for Tuberculosis and the European Tuberculosis Surveillance Network.

Terms (verbatim definitions)

Migrant: *An umbrella term, not defined under international law, reflecting the common lay understanding of a person who moves away from his or her place of usual residence, whether within a country or across an international border, temporarily or permanently, and for a variety of reasons.* Source: IOM glossary(2)

Asylum-Seeker: *An asylum-seeker is an individual who is seeking international protection. In countries with individualized procedures, an asylum-seeker is someone whose claim has not yet been finally decided on by the country in which he or she has submitted it. Not every asylum- seeker will ultimately be recognized as a refugee, but every refugee is initially an asylum-seeker.* Source: UNHCR Master Glossary of Terms (2006)(3).

Immigration status: *The status of a migrant under the immigration law of the country of destination.* Source IOM glossary(2)

Refugee: *A person who meets the eligibility criteria under the applicable refugee definition, as provided for in international or regional refugee instruments, under UNHCR's mandate, and/or in national legislation.* Source: UNHCR Master Glossary of Terms (2006)(3).

Immigrant: *From the perspective of the country of arrival, a person who moves into a country other than that of his or her nationality or usual residence, so that the country of destination effectively becomes his or her new country of usual residence.* Source: IOM glossary(2)

Additional tables and figures

	Multivariate Analysis			
	**aOR**	**p Value**	**95% Confidence interval**	
**male sex**	1.08	0.15	0.97	1.21
**age group**				
<18 years old	0.36	<0.001	0.29	0.44
18-34 years old	reference group			
35-54 years old	0.89	<0.001	0.76	1.04
55+ years old	2.90	<0.001	2.37	3.55
**Migrant typology**				
UK Students and workers	reference group			
NL immigrants	1.14	0.26	0.91	1.42
Asylum seekers	4.62	<0.001	3.83	5.58
UK settlements and family	1.77	<0.001	1.56	2.01
UK working holiday and others	1.05	0.68	0.82	1.36
**TB contact/ Incidence in country of origin**				
No TB contact/ Incidence less than 50	reference group			
No TB contact/ Incidence between 50 and 100	2.07	<0.001	1.41	3.03
No TB contact/ Incidence between 100 and 200	7.50	<0.001	5.28	10.66
No TB contact/ Incidence between 200 and 300	6.55	<0.001	4.59	9.35
No TB contact/ Incidence more than 300	20.24	<0.001	14.18	28.88
TB contact/ Incidence less than 50	1.00			
TB contact/ Incidence between 50 and 100	382.24	<0.001	114.13	1280.18
TB contact/ Incidence between 100 and 200	92.64	<0.001	40.53	211.74
TB contact/ Incidence between 200 and 300	96.52	<0.001	53.81	173.13
TB contact/ Incidence more than 300	152.09	<0.001	76.18	303.63
**time period of screening**				
before 2010	reference group			
2010-2012	1.28	0.00	1.08	1.52
2013-2015	1.57	<0.001	1.34	1.85
2016 and beyond	0.77	0.00	0.65	0.92

Annex table S1: logistic regression model assessing predictors for TB at the time of screening (prevalent TB or yield) fitting interaction terms between TB contact and country of origin. LR Test p<0.003 (comparing this model to one without interaction terms)

	aOR	p Value	95% CI	
**male sex**	1.07	0.22	0.96	1.19
**Age/Migrant type**				
NL Immigrants/<18 years	0.39	0.06	0.15	1.06
NL Immigrants/18-35 years	1.16	0.22	0.91	1.48
NL Immigrants/35-54 years	0.64	0.17	0.34	1.20
NL Immigrants/55+ years	2.83	0.14	0.70	11.40
Asylum seekers/<18 years	2.31	<0.001	1.66	3.22
Asylum seekers/18-35 years	4.52	<0.001	3.62	5.64
Asylum seekers/35-54 years	3.49	<0.001	2.33	5.24
Asylum seekers/55+ years	3.14	0.02	1.17	8.45
UK settlement& family/<18 years	0.49	<0.001	0.36	0.66
UK settlement& family/18-35 years	1.51	<0.001	1.31	1.75
UK settlement& family/35-54 years	1.98	<0.001	1.61	2.43
UK settlement& family/55+ years	6.40	<0.001	5.13	7.97
UK student & work/<18 years	0.36	0.00	0.18	0.72
UK student & work/18-35 years	reference group			
UK student & work/35-54 years	0.51	<0.001	0.35	0.74
UK student & work/55+ years	0.89	0.87	0.22	3.56
UK Working Holiday & other/<18 years	0.13	0.04	0.02	0.94
UK Working Holiday & other/18-35 years	1.19	0.25	0.89	1.60
UK Working Holiday & other/35-54 years	0.92	0.78	0.53	1.61
UK Working Holiday & other/55+ years	1.45	0.37	0.65	3.25
**Incidence in country of origin**				
Less than 50	reference group			
Between 50 and 100	2.03	<0.001	1.39	2.98
Between 100 and 200	7.30	<0.001	5.13	10.38
Between 200 and 300	6.36	<0.001	4.46	9.08
More than 300	19.04	<0.001	13.34	27.18
**Contact with TB case**	11.99	<0.001	8.66	16.60
**time period of screening**				
before 2010	reference group			
2010-2012	1.30	0.00	1.10	1.54
2013-2015	1.59	<0.001	1.35	1.87
2016 and beyond	0.78	0.01	0.66	0.94

Annex table S2: logistic regression model assessing predictors for TB at the time of screening (prevalent TB or yield) fitting interaction terms between age and migrant typology. LR Test p<0.001 (comparing this model to one without interaction terms)

	**Univariate analysis**				**Multivariate Analysis**				
	**OR**	**p Value (Wald)**	**95% CI**		**aOR**	**p Value (Wald)**	**95% CI**		**LR Test**
**male sex**	0.92	0.15	0.82	1.03	1.08	0.24	0.95	1.23	0.15
**age group**									
**<18 years old**	2.55	<0.001	1.97	3.32	3.85	<0.001	2.60	5.71	
**18-34 years old**	reference category								<0.001
**35-54 years old**	2.35	<0.001	1.74	3.18	3.12	<0.001	2.02	4.82	
**55+ years old**	3.26	<0.001	2.38	4.47	12.07	<0.001	6.48	22.46	
**Migrant typology**									
**UK Students and workers**	reference category								<0.001
**NL immigrants**	0.43	<0.001	0.33	0.55	0.24	<0.001	0.16	0.37	
**Asylum seekers (IT, NL, SE)**	1.04	0.68	0.86	1.27	1.12	0.73	0.59	2.12	
**UK settlements and family**	0.48	<0.001	0.42	0.54	0.56	<0.001	0.47	0.66	
**UK working holiday and others**	0.45	<0.001	0.33	0.61	0.44	<0.001	0.43	0.45	
**Incidence in country of origin**									
**Less than 50**	reference category								<0.001
**Between 50 and 100**	0.83	0.40	0.55	1.27	1.64	0.30	0.64	4.21	
**Between 100 and 200**	3.61	<0.001	2.47	5.27	7.79	<0.001	5.57	10.91	
**Between 200 and 300**	3.50	<0.001	2.41	5.08	7.02	<0.001	5.18	9.51	
**More than 300**	4.58	<0.001	3.11	6.74	15.58	<0.001	12.42	19.54	
**tbcontact2**									
**no**	reference category								
**yes**	20.40	<0.001	14.32	29.06	11.66	<0.001	11.62	11.70	<0.001
**unknown**	1.22	0.01	1.06	1.40	2.13	0.01	1.24	3.66	
**time period of screening**									
**before 2010**	reference category								<0.001
**2010-2012**	1.94	<0.001	1.58	2.38	1.94	<0.001	1.53	2.46	
**2013-2015**	1.70	<0.001	1.41	2.05	2.35	<0.001	2.30	2.39	
**2016 and beyond**	1.14	0.17	0.94	1.38	1.49	0.04	1.01	2.20	

Annex table S3: logistic regression model assessing predictors for culture-confirmed TB at the time of screening. Standard errors were adjusted for clustering at the programme level. TB Contacts are either family or other close (household type) contacts. Incidence in country of origin refers to 2019 WHO estimates. P-values are calculated using the likelihood ratio test comparing to restricted models with less predictors. OR: Odds Ratios, aOR: adjusted Odds Ratios, CI: confidence intervals.

	Univariate analysis				Multivariate Analysis				
	OR	p Value (Wald)	95% CI		aOR	p Value (Wald)	95% CI		LR Test
**male sex**	0.90	<0.001	0.89	0.92	0.92	0.29	0.79	1.08	<0.001
**age group**									
<18 years old	1.17	<0.001	1.14	1.20	1.38	<0.001	1.23	1.55	
18-34 years old	reference category								<0.001
35-54 years old	2.23	<0.001	2.17	2.30	2.46	<0.001	1.98	3.05	
55+ years old	9.86	<0.001	9.51	10.21	10.66	<0.001	6.96	16.33	
**Migrant typology**									
UK Students and workers	reference category								<0.001
NL immigrants	1.58	<0.001	1.54	1.61	1.00	0.85	0.96	1.05	
Asylum seekers	1.21	<0.001	1.18	1.24	0.86	<0.001	0.80	0.93	
UK settlements and family	0.57	<0.001	0.57	0.58	0.80	0.03	0.65	0.97	
UK working holiday and others	1.07	<0.001	1.04	1.10	1.01	0.83	0.95	1.06	
**Incidence in country of origin**									
Less than 50	reference category								<0.001
Between 50 and 100	0.34	<0.001	0.33	0.35	0.62	0.39	0.21	1.84	
Between 100 and 200	0.89	<0.001	0.87	0.91	1.38	<0.001	1.19	1.59	
Between 200 and 300	0.71	<0.001	0.69	0.73	1.11	0.55	0.78	1.58	
More than 300	1.41	<0.001	1.37	1.45	1.91	<0.001	1.58	2.31	
**tbcontact2**									
no	reference category								
yes	4.98	<0.001	4.53	5.47	3.98	<0.001	3.33	4.76	<0.001
unknown	1.99	<0.001	1.96	2.02	2.26	<0.001	2.14	2.38	
**time period of screening**									
before 2010	reference category								<0.001
2010-2012	0.92	<0.001	0.90	0.94	0.72	0.01	0.56	0.92	
2013-2015	0.80	<0.001	0.79	0.81	0.58	<0.001	0.55	0.61	
2016 and beyond	0.64	<0.001	0.63	0.65	0.56	<0.001	0.49	0.63	

Annex table S4: logistic regression model assessing predictors for TB-related CXR abnormality at the time of screening. Standard errors were adjusted for clustering at the programme level. TB Contacts are either family or other close (household type) contacts. Incidence in country of origin refers to 2019 WHO estimates. P-values are calculated using the likelihood ratio test comparing to restricted models with less predictors. OR: Odds Ratios, aOR: adjusted Odds Ratios, CI: confidence intervals.

	aOR	p Value (Wald)	95% Confidence interval	
**male sex**	1.05	0.39	0.94	1.17
**age group**				
<18 years old	0.35	<0.01	0.28	0.43
18-34 years old	reference category			
35-54 years old	0.88	0.10	0.75	1.02
55+ years old	2.85	<0.01	2.32	3.49
**Migrant type/ Incidence in country of origin**				
NL immigrants/ Incidence less than 50	0.45	0.17	0.14	1.39
NL immigrants/ incidence between 50 and 100	0.69	0.48	0.25	1.92
NL immigrants/ incidence between 100 and 200	1.61	0.33	0.62	4.19
NL immigrants/ incidence between 200 and 300	4.98	<0.01	1.81	13.69
NL immigrants/ incidence more than 300	6.20	<0.01	2.49	15.47
Asylum seekers/ Incidence less than 50	1.14	0.79	0.44	2.94
Asylum seekers/ incidence between 50 and 100	5.76	<0.01	2.32	14.29
Asylum seekers/ incidence between 100 and 200	9.33	<0.01	3.78	23.07
Asylum seekers/ incidence between 200 and 300	9.98	<0.01	3.96	25.17
Asylum seekers/ incidence more than 300	9.64	<0.01	3.54	26.25
UK settlement& family/ Incidence less than 50	1.00*			
UK settlement& family/ incidence between 50 and 100	1.37	0.52	0.53	3.57
UK settlement& family/ incidence between 100 and 200	5.76	<0.01	2.54	13.03
UK settlement& family/ incidence between 200 and 300	4.27	<0.01	1.90	9.61
UK settlement& family/ incidence more than 300	17.42	<0.01	7.67	39.59
UK student & work/ Incidence less than 50	reference category			
UK student & work/ incidence between 50 and 100	0.50	0.12	0.21	1.20
UK student & work/ incidence between 100 and 200	3.48	<0.01	1.54	7.88
UK student & work/ incidence between 200 and 300	2.82	0.01	1.25	6.38
UK student & work/ incidence more than 300	6.67	<0.01	2.90	15.34
UK Working Holiday/ Incidence less than 50	1.00*			
UK Working Holiday/ incidence between 50 and 100	0.89	0.85	0.27	2.93
UK Working Holiday/ incidence between 100 and 200	0.68	0.52	0.21	2.23
UK Working Holiday/ incidence between 200 and 300	3.45	0.01	1.41	8.44
UK Working Holiday/ incidence more than 300	14.95	<0.01	6.26	35.71
**Contact of TB case**				
no	reference category			

yes	12.15	<0.01	8.76	16.84
unknown	1.49	0.02	1.06	2.08
**time period of screening**				
before 2010	reference group			
2010-2012	1.27	0.01	1.08	1.51
2013-2015	1.53	<0.01	1.29	1.80
2016 and beyond	0.77	0.01	0.64	0.92

Annex Table S5: logistic regression model assessing determining factors for prevalent TB at the time of screening (yield) with interaction terms for migrant typology and country of origin; standard errors adjusted for clustering at the programme level. Interaction terms are presented as TB yield in each stratum. NL: The Netherlands, UK: United Kingdom, *perfectly predicts failure (small cell volume)

References

1. European Commission. Commission Implementing Decision (EU) 2018/945 of 22 June 2018 on the communicable diseases and related special health issues to be covered by epidemiological surveillance as well as relevant case definitions (Text with EEA relevance.) [Internet]. OJ L, 32018D0945 Jul 6, 2018. Available from: http://data.europa.eu/eli/dec_impl/2018/945/oj/eng

2. International Organization for Migration. Glossary on Migration (2019) [Internet]. 2016 Nov [cited 2021 Feb 26]. Available from: https://www.iom.int/glossary-migration-2019

3. United Nations High Commissioner for Refugees. UNHCR Master Glossary of Terms [Internet]. [cited 2021 Feb 26]. Available from: https://www.refworld.org/docid/42ce7d444.html
