# Supplementary material for: Drivers determining tuberculosis disease screening yield in four European screening programmes: a comparative analysis
Source: Eur Respir J. 2023 Oct 12;62(4):2202396. doi: 10.1183/13993003.02396-2022 (PMC10568038; doi:10.1183/13993003.02396-2022)
Supplement: Supplementary file 2 [file ERJ-02396-2022.Shareable.pdf]

EUROPEAN RESPIRATORY JOURNAL
ORIGINAL RESEARCH ARTICLE
D. ZENNER ET AL.

SHAREABLE PDF

Drivers determining tuberculosis disease screening yield in four European screening programmes: a comparative analysis

Dominik Zenner[1,2,3,4], Daniella Brals[3,4], Joanna Nederby-Öhd[5], Dee Menezes[6], Robert Aldridge[6], Sarah R. Anderson[7], Gerard de Vries[8], Connie Erkens[9], Valentina Marchese[10], Alberto Matteelli[10], Morris Muzyamba[7], Job van Rest[9], Ineke Spruijt[9], John Were[1], Giovanni Battista Migliori[11], Knut Lönnroth[5], Frank Cobelens[3,4] and Ibrahim Abubakar[1]

[1]Faculty of Population Health Sciences, University College London, London, UK. [2]Wolfson Institute of Population Health, Queen Mary University of London, London, UK. [3]Amsterdam University Medical Centers, location University of Amsterdam, Department of Global Health, Amsterdam, The Netherlands. [4]Amsterdam Public Health, Global Health, Amsterdam, The Netherlands. [5]Department of Global Public Health, Karolinska Institutet, Stockholm, Sweden. [6]Institute of Health Informatics Research, University College London, London, UK. [7]UK Health Security Agency, London, UK. [8]National Institute for Public Health and the Environment, Bilthoven, The Netherlands. [9]KNCV Tuberculosis Foundation, The Hague, The Netherlands. [10]WHO Collaborating Center for TB/HIV and the TB Elimination Strategy, University of Brescia, Brescia, Italy. [11]Servizio di Epidemiologia Clinica delle Malattie Respiratorie, Istituti Clinici Scientifici Maugeri IRCCS, Tradate, Italy.

Corresponding author: Dominik Zenner (d.zenner@qmul.ac.uk)

Check for updates

Shareable abstract (@ERSpublications)
Factors associated with TB screening yield included increasing age, migrant typology, TB incidence in country of origin, TB case contact and period of screening. The TB yield among asylum seekers was higher than for other migrant categories. https://bit.ly/3VMLwFp

Cite this article as: Zenner D, Brals D, Nederby-Öhd J, *et al*. Drivers determining tuberculosis disease screening yield in four European screening programmes: a comparative analysis. *Eur Respir J* 2023; 62: 2202396 [DOI: 10.1183/13993003.02396-2022].

This extracted version can be shared freely online.

Copyright ©The authors 2023.

This version is distributed under the terms of the Creative Commons Attribution Licence 4.0.

This article has an editorial commentary: https://doi.org/10.1183/13993003.01537-2023

Received: 12 Dec 2022
Accepted: 3 May 2023

Abstract

Background The World Health Organization End TB Strategy emphasises screening for early diagnosis of tuberculosis (TB) in high-risk groups, including migrants. We analysed key drivers of TB yield differences in four large migrant TB screening programmes to inform TB control planning and feasibility of a European approach.

Methods We pooled individual TB screening episode data from Italy, the Netherlands, Sweden and the UK, and analysed predictors and interactions for TB case yield using multivariable logistic regression models.

Results Between 2005 and 2018 in 2 302 260 screening episodes among 2 107 016 migrants to four countries, the programmes identified 1658 TB cases (yield 72.0 (95% CI 68.6–75.6) per 100 000). In logistic regression analysis, we found associations between TB screening yield and age (⩾55 years: OR 2.91 (95% CI 2.24–3.78)), being an asylum seeker (OR 3.19 (95% CI 1.03–9.83)) or on a settlement visa (OR 1.78 (95% CI 1.57–2.01)), close TB contact (OR 12.25 (95% CI 11.73–12.79)) and higher TB incidence in the country of origin. We demonstrated interactions between migrant typology and age, as well as country of origin. For asylum seekers, the elevated TB risk remained similar above country of origin incidence thresholds of 100 per 100 000.

Conclusions Key determinants of TB yield included close contact, increasing age, incidence in country of origin and specific migrant groups, including asylum seekers and refugees. For most migrants such as UK students and workers, TB yield significantly increased with levels of incidence in the country of origin. The high, country of origin-independent TB risk in asylum seekers above a 100 per 100 000 threshold could reflect higher transmission and re-activation risk of migration routes, with implications for selecting populations for TB screening.
